# Functional alignment is a feasible alignment strategy in robotic assisted total knee arthroplasty for knee osteoarthritis with extra-articular deformity – A case series

**DOI:** 10.1051/sicotj/2024059

**Published:** 2025-01-13

**Authors:** Wei Cheong Eu, Jade Pei Yuik Ho, G. Kunalan

**Affiliations:** Department of Orthopaedic Surgery, Joint Replacement Unit, Kuala Lumpur Hospital, Ministry of Health Malaysia Jalan Pahang 50586 Kuala Lumpur Malaysia

**Keywords:** Extraarticular deformity, Functional alignment, Robotic assisted total knee arthroplasty

## Abstract

*Introduction*: Extraarticular deformity (EAD) with knee arthritis is a complex condition involving tri-planar bone deformity with pathological malalignment and chronic soft tissue contracture or laxity in the knee joint. Intraarticular correction by TKA, which was previously performed with conventional manual jig by mechanical alignment technique, had its limits and difficulties especially extensive soft tissue release and risk of jeopardizing the collateral ligaments. Robotic technology allows for reproducible and precise execution of surgical plan and allows adjustment to various new personalised alignment philosophy including functional alignment (FA). FA technique involves the adjustment of components positioning that least compromise the soft tissue envelope while restoring the limb alignment and joint obliquity to create a balanced knee. The aim is to study the outcome of intra-articular correction by robotic assisted TKA using Functional Alignment (FA) technique. *Methodology*: This is a single surgeon series of 8 patients with extraarticular deformity who underwent robotic assisted total knee arthroplasty (TKA) with FA technique. Soft tissue release was gradually released and followed by adjustments of implant positioning in order to achieve a balanced medio-lateral gap. *Results*: Postoperatively, the lower limb alignment of all patients were restored within 6° (mean 4.54°) based on functional alignment boundaries. Knee phenotype and joint line obliquity (JLO) were restored in comparison to contralateral lower limb. There were 6 varus and 2 valgus malalignment. 7 patients were implanted with posterior stabilized implants while 1 was implanted with cruciate retaining implant. Arc of knee flexion and extension improved (*P* = 0.002). There was a large postoperative improvement in the Knee Society Score (KSS) (*P* < 0.001). *Discussion*: Intraarticular correction by TKA for EAD with knee arthritis is technically reliable with robotic technology. It allows intraoperative adjustment following functional alignment philosophy, thereby, restoring pre-arthritic alignment, knee phenotype and joint line obliquity.

## Introduction

Long bone fracture malunion is one of the many causes of extra-articular deformity (EAD). The deformity causes deviation of weight bearing axis and pathological loading which results in early knee osteoarthritis. EAD with knee osteoarthritis is very rare nowadays as most long bone fractures are being treated surgically with proper reduction. There are 3 main options in managing EAD with knee arthritis: (1) joint resurfacing omitting the EAD, (2) hybrid technique of partially compensating the deformity by TKA intra-articular correction and (3) corrective osteotomy combined with TKA (simultaneous or staged) [1].

Intraarticular correction by TKA alone for EAD were previously performed following mechanical alignment concept and well described by Wang et al. [2]. However, mechanical alignment TKA in EAD had its limits, including extensive soft tissue release and risk of bone cuts breaching the collateral ligaments and necessitating a constrained implant. Few studies reported TKA with mechanical alignment (0°) failed to reproduce native anatomy and joint kinematics [3, 4]. In addition, it is challenging to perform the surgery with conventional intramedullary jig method as the canal was possibly sclerotic and might be obliterated with metal implant. There is also lack of reproducible precision in bone cut and alignment correction with conventional method. Navigation system was previously used for this reason and most recently, the advent of robotic assisted technology gives the advantages of accurate bone cuts and precise execution of various new personalized alignment strategies including kinematic alignment (KA), restricted kinematic alignment (rKA), inverse kinematic alignment (iKA) and functional alignment (FA). FA is an evolution of kinematic alignment with bone resections, soft tissue releases and implant positioning adjusted within a safe zone depending on a patient’s native alignment [5].

The objective of this write up is to demonstrate the feasibility of intraarticular correction by robotic assisted TKA in knee arthritis with EAD and to describe the use of functional alignment technique in restoring limb alignment while maintaining the knee phenotypes and joint surface orientation.

## Materials and methods

This is a retrospective analysis of prospective collected data including 8 patients’ record from the year 2022–2024. Patients with the diagnosis of knee osteoarthritis with extra-articular deformity (EAD) secondary to fracture malunion were included. All cases have tri-planar deformity. Ethical approval was obtained from National Medical Research Register committee (NMRR ID-24-00568-FE9).

### Preoperative clinical assessment and planning

All patients were examined preoperative for medial and lateral collateral ligament status, rotational profile and range of movement (ROM). Patients with rotational profile less than 10° difference were recruited. Preoperative and postoperative bilateral long limb radiograph were performed. Hip knee angle (HKA), long bone malunion deformity angle in coronal and sagittal plane, tibia slope, joint line orientation angle (JLOA), lateral distal femoral angle (LDFA) and medial proximal tibial angle (MPTA) and joint line obliquity were measured. The long bone malunion deformity angle represented the severity of the EAD and was measured as the angle subtended by anatomical axis of proximal and distal part of long bone at the malunion site in both coronal and sagittal plane. Both lower limb knee phenotypes were determined based on Coronal Plane Knee Phenotypes (CPAK) classification. For femoral deformity, a perpendicular line was drawn to the mechanical axis of femur as a proposed distal femur cut. If this exceeded the collateral ligament attachment, an osteotomy would be considered. Similarly, for tibia deformity, a perpendicular line was drawn perpendicular to distal tibia axis and if the proposed tibia cut breached the attachment of collateral ligament, an osteotomy would be considered.

### Surgical technique

All surgeries were performed by single surgeon using fully cemented knee prosthesis (Persona, Zimmer Biomet, Warsaw, Indiana, USA) with the assistance of robotic technology (ROSA Zimmer Biomet). Medial parapatellar approach and posterior cruciate ligament release was performed for all cases. Navigation tracker pins were inserted followed by registration. Preoperative ROM, HKA and medial-lateral gaps were examined with feedback from robotic system. Varus and valgus stress were applied at 0°, 45° and 90° knee flexion.

Surgical technique was performed following FA philosophy [5–7]. The starting point was from mechanical standpoint with femur and tibia set at 0°. Soft tissue release and osteophytes removal were first performed step-wise gradually at surgeon discretion with the aim of correcting the pathological alignment closer to native limb alignment. HKA angle was then assessed after soft tissue release. Next, the femoral and tibia implant positioning and bone cuts were adjusted based on HKA correction after soft tissue release to attain an equal and balanced 19 mm medio-lateral gap in extension, which was the combined thickness of the implant. The bone cuts should not breach through the collateral ligaments. In our cases, femur cut was performed first followed by tibia cut.

### Varus prototype

In the coronal plane, the femur cut ranged from 6° valgus to 3° varus. Femoral sagittal flexion was preset up to 5° to avoid anterior femur notching. In the coronal plane, tibia component positioning was limited within 0° valgus to 6° varus. Tibia slope was limited to within 3°. Extension gap was then checked with spacer block. The aim was to correct the pre-operative HKA within 0° valgus to 6° varus for the varus prototype. If the HKA was beyond the targeted range, the adjustment of cuts would be made based on LDFA or MPTA values whichever contributed the most to the deformity.

In the axial plane, rotation of femoral component was set within 3° IR to 6° ER from trans-epicondylar axis by using FuZion^TM^ device with tension load being applied. Flexion gap was targeted at 20 mm/20 mm medio-laterally and within 1 mm difference. Trial of implant was performed followed by range of motion (ROM) and gap balance assessment. Patella tracking or patella-femoral articulation was then assessed by ‘no thumb technique’ throughout the flexion arc with the torniquet being deflated. Lateral retinaculum release (inside-out technique) was performed at approximately 1 cm lateral-to-lateral margin of patella when necessary.

### Valgus prototype

In the coronal plane, femur component positioning was adjusted within 6° mechanical valgus. For the tibia component positioning, the target was within 2° valgus. Our aim was to restore the final alignment within 3° valgus (183°) and maintain the pre-arthritic knee phenotype. Other adjustment limits were similar to the FA technique as reported by Jobe Shatrov et al. [7]. For the lateral soft tissue contracture in valgus knee, the posterolateral capsule and iliotibial band were released in a stepwise fashion by pie crusting technique using Blade size 15 as described by Ranawat et al in order to achieve a balanced extension gap [8].

### Postoperative assessment and outcome measurment

All patients had bilateral long limb radiograph performed at day-2 post surgery. Patients were followed up in clinic at week 2, 6, 12 and then 6 monthly. The primary outcome was to restore pre-arthritic limb alignment and knee phenotype based on the CPAK knee classification. The outcome measures include HKA angle, joint line obliquity and knee phenotype. The secondary outcome was to assess the improvement in knee function after surgery. ROM and Knee Society Score (KSS) was assessed before and 12-month after surgery. Data analysis was performed with SPSS version 29.0.2 (IBM SPSS Statistics for Windows, IBM Corp., Armonk, NY, USA) using student *t*-test. The measurements were performed by fellowship trained hip and knee arthroplasty subspecialty surgeons who were the first and second authors.

## Result

The mean period of follow up was 18 months (12–26 months). The average age of the patients was 69.8 years. The average Body Mass Index (BMI) was 28 kg/m^2^ (Table 1). The mean coronal deformity angle at femur was 18.8° varus (*n* = 4) with one valgus deformity of 15° whereas the mean coronal deformity at tibia was 11.2° (*n* = 3) with one valgus of 8.3°. The mean sagittal deformity of femur was 12.3° procurvatum (*n* = 4) and only one recurvatum at 14.5°. For tibia sagittal deformity, the mean was 6.7° for procurvatum type (*n* = 2) and only one recurvatum at 5.7°.

**Table 1:** Demographic of patients and clinical outcome.^a^

No	Age/Sex	BMI Kg/m^2^	Cause of deformity	Site	Deformity (°)				Alignment	Preop ROM (°)	Postop ROM (°)	Preop KSS	Preop function score	Postop KSS	Postop function score	F/U (years)	Comp
					Coronal femur	Sagittal femur	Coronal tibia	Sagittal tibia									
1	70/M	30	Malunion	Femur	18	14.7			Varus Procurvatum	10–80	0–100	38	0	93	80	1.8	No
2	77/M	24	Malunion with plate in-situ	Femur	15	6.9			Valgus Procurvatum	20–100	5–100	25	30	87	85	2.2	No
3	70/M	30	Malunion	Femur	23	14.5			Varus Recurvatum	5–40	0–85	17	30	87	85	2	No
4	66/M	25.5	Malunion	Femur	15	18.6			Varus Procurvatum	10–75	0–100	25	30	95	80	1.5	Knee Hematoma
5	59/M	30	Malunion	Femur	19.1	9.1			Varus Procurvatum	10–90	5–100	39	45	75	70	1	No
6	71/F	25	Malunion	Tibia			8.3	5.7	Valgus Recurvatum	10–120	0–120	30	0	89	55	1	No
7	73/M	34	Malunion	Tibia			10	8	Varus Procurvatum	10–110	0–110	35	30	86	90	1.2	No
8	72/M	23	Malunion	Tibia			12.4	5.4	Varus Procurvatum	10–110	0–110	38	45	86	90	1.3	No

a KSS = Knee Society Score, ROM = Range of motion, BMI = Body Mass Index, F/U = Follow up, Comp = Complication.

All surgeries were performed solely with intraarticular correction without osteotomy. 7 out of the eight was implanted with Posterior Stabilized (PS) knee implant and only one had Cruciate Retaining (CR) implant with ultra-congruent (UC) polyethylene. 6 out of 8 patients required lateral retinaculum release and patella lateral facetectomy for patella mal-tracking intraoperatively due to chronic lateral soft tissue contracture from malalignment. All patients had normal patella tracking during follow up.

### Primary outcome

**Figure 1:**
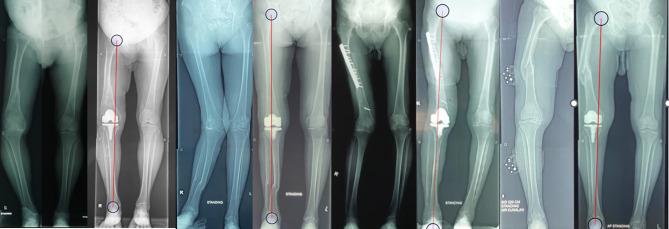
Preoperative Scannogram and Postoperative Scannogram (Radiograph showing long limb Anterior-Posterior view of four patients with red colour line corresponding to lower limb axis from center of hip joint to center of ankle joint).

The mean preoperative HKA angle was 17.6° varus (*n* = 6) and improved to 5.2° varus postoperatively (*P* < 0.001). For valgus malalignment, the mean preoperative HKA angle was 21° and corrected to 2.5° postoperatively (Table 2) within 3° valgus limit in the valgus prototype FA criteria [7]. The overall correction angle was mean 18.5° preoperative to 4.5° postoperative (*P* < 0.001). Lower limb axis was corrected closer to the native limb axis as shown in Figure 1.

**Table 2:** Comparison of preoperative and postoperative outcomes^c^ (*n* = 8).

	Preoperative		Postoperative		*P-*value
	Mean	SD	Mean	SD	
Range of motion (°)					
Knee flexion	83.13	20.17	103.13	10.33	0.002
Knee extension	11.25	3.54	1.25	2.32	<0.001
					
Knee society score (points)					
Knee	30.88	7.99	87.25	5.97	<0.001
Function	26.25	17.47	79.38	11.78	<0.001
					
HKA angle (°)					
Varus (*n* = 6)	17.63	3.61	5.23	1.68	<0.001
Valgus (*n* = 2)	21.00	7.21	2.45	1.2	0.198
Overall total correction all patients (*n* = 8)	18.48	4.38	4.54	1.97	<0.001

c SD = Standard Deviation, ° = Degree.

*P*-value determined by Paired-Samples *t*-test.

The mean LDFA was 93.1° (range from 77.8 to 104.8°) and was corrected after surgery to mean 91.5° (range from 87.5° to 94.0°). The mean MPTA pre-operation was 84.5° and corrected to 88.2° (Table 3). The mean tibia slope before surgery was 11.2° and reduced to 3.3° after surgery. All patients’ knee phenotype based on CPAK classification were restored. Joint line obliquity (JLO) was also maintained in comparison to contralateral lower limb.

**Table 3:** Radiological measurement of Alignment, Joint Line Obliquity and Knee Phenotypes.^b^

No	Type of implant during surgery	Preop HKA angle	Postop HKA angle	Preop LDFA	Coronal alignment of femoral component (Post-op LDFA)	Preop MPTA	Coronal alignment of tibia component (Post-op MPTA)	JLOA preop	JLOA post op	JLO post op	JLO contralateral limb	CPAK Pre-op/Post-op	Tibial slope preop	Tibial slope postop	Intra-op patella maltracking
1	PS	14.0	4.0	100.4	92.5	86.6	90.0	4.2	2.4	AP	AP	Ⅶ/Ⅶ	10.0	3.0	Yes
2	PS	15.9	3.3	77.8	87.5	92.6	91.0	4.9	5.1	AD	AD	Ⅲ/Ⅲ	11.0	2.0	Yes
3	PS	22.7	5.8	104.8	94.0	85.8	88.2	−7.0	−0.5	AP	AP	Ⅶ/Ⅶ	10.0	1.0	Yes
4	PS	19.5	5.9	100.1	92.9	83.0	86.9	−6.3	0.0	N	N	Ⅳ/Ⅳ	10.0	3.0	Yes
5	PS	20.1	7.4	96.3	93.4	76.9	86.6	6.5	2.3	AD	AD	Ⅰ/Ⅰ	12.0	0.2	Yes
6	PS	26.1	1.6	80.8	89.1	93.7	91.7	10.1	1.0	AD	AD	Ⅲ/Ⅲ	6.7	3.3	Yes
7	PS	14.5	2.6	91.5	89.8	81.5	88.0	2.0	3.0	AD	AD	Ⅰ/Ⅰ	10.0	7.0	No
8	CR/UC insert	15.0	5.7	93.4	91.2	76.1	85.0	3.0	1.8	AD	AD	Ⅰ/Ⅰ	20.0	7.0	No

b HKA angle = Hip-Knee-Ankle angle, LDFA = Lateral Distal Femoral Angle, MPTA = Medial Proximal Tibial Angle, JLOA = Joint Line Obliquity Angle, JLO = Joint Line Obliquity based on Coronal Plane Alignment Knee (CPAK) Classification, AP = Apex Proximal, AD = Apex Distal, N = Neutral, Roman numerals Ⅰ–Ⅸ represent knee phenotypes.

### Secondary outcome

For the ROM assessment, the most severe flexion contracture was 20° preoperatively with a mean of 11.3°. The mean knee extension after surgery was 1.25° which improved significantly (*P* < 0.001). Knee flexion arc had also improved from mean 83.1 to 103.1° (Table 2). The worst range recorded was 5–40° pre-operation and improved to 0–85°.

Other secondary outcomes including the knee function, which was assessed by Knee Society Score (KSS). The mean knee score pre-operation was 30.9 points and subsequently improved to 87.3 points post-surgery. The mean functional score was 26.3 points and improved to 79.4 points after TKA (Table 2).

## Discussion

This is a case series to report that functional alignment robotic TKA could be performed in EAD with knee arthritis, which show good result in the short term [9]. In the past, multiple studies have shown the benefits of using computer navigation in this complex surgery [10–12].

There were three main options for management of EAD: (1) joint resurfacing and omitting the EAD, (2) intra-articular correction by TKA to partially compensate for the deformity and (3) deformity correction by osteotomy followed by TKA either performed as staged or simultaneous surgery [1]. Corrective osteotomy has its disadvantages which include longer operative time or double surgery, delay in weightbearing, non-union at osteotomy site, joint stiffness and larger incision wound with infection Lonner et al reported complication rate up to 45% with non-union and joint stiffness in their series [13].

Intra-articular correction by TKA or lately described as ‘hybrid technique’ is still possible with the many published studies showing good results [1]. It can be performed by conventional manual technique or by newer advent robotic assisted surgery. Wang et al concluded that intra-articular correction is feasible for EAD of 20° coronal plane in the femur and 30° in the tibia. The magnitude of deformity and distance of deformity to the knee joint affects the possibility of intra-articular correction [14]. On the contrary, up to 15 degrees of procurvatum or recurvatum in the sagittal plane could be considered for TKA [15].

It offers advantages of single surgery with immediate weight bearing, earlier pain relief and functional recovery, shorter hospital stay with single smaller wound and avoid complications related to osteotomy. This is especially suitable for elderly patient with low demand with shorter recovery time and faster rehabilitation [16, 17].

Patient with fracture malunion in femur or tibia has changes in the anatomical axis and deviation in the mechanical axis, which affects the knee joint kinematics. The surgery is complex and needs proper planning even in the experienced hand. Conventional TKA method using intramedullary guide is difficult due to canal sclerosis and obstruction by the presence of retained hardware. It is also associated with more outliers in alignment and implant positioning and difficult to achieve a well balance knee in this complex scenario [18].

Robotic surgery offers intraoperative feedback on gap balancing with corresponding final alignment especially in such complex surgery. The cut precision accuracy is up to 0.7 mm and error up to 1°. It allows surgeon to adjust the cuts within safe zone following various alignment strategy and avoid breaching the collateral ligaments. Implant position can be tailored based on individual anatomy and ligament tensioning [19, 20]. Patient with EAD have excessive tibial slope as shown in Table 3, which together with PCL and posterior capsule laxity could result in a loose flexion gap and subsequently instability. With robot, this concern on gap balancing would be addressed intraoperatively with pre-set reduced tibia slope and adjustments of bone cuts.

There are various alignment philosophy evolving from mechanical alignment (MA), kinematic alignment (KA) to Functional alignment (FA). The CPAK classification reported 26.4% of varus knee and 39.2% with neutral alignment has apex distal or oblique joint line. The study reported KA TKA group has better gap balance as measured by pressure sensor [21].

Patient with EAD with malalignment had its own learned and adapted functional ligament laxity pattern. Correcting the final alignment to mechanical alignment (MA) may cause unnatural knee feelings. Newer kinematic alignment (KA) philosophy including the latest Functional Alignment (FA) strategy aims to restore knee kinematics as close to natural. Knee laxity is tighter at 0° of flexion than at 45° and 90° of flexion. KA TKA was shown to have better clinical outcomes and higher satisfaction compared to MA TKA [22–24]. The 10-year implant survivorship with kinematic aligned knee is at 97.5% revision for any reason and 98.4% for aseptic failure [25]. It was explained that the joint line was more parallel to floor in kinematic aligned TKA and it prevents overload of medial or lateral compartment and therefore prevents early failure [23, 26]. However, kinematic alignment is not feasible to every patient due to large variability in individual anatomy and limb alignment with no clear limits on its surgical planning.

The aim of intra-articular correction or ‘hybrid technique’ is to bring the lower limb axis as close to native axis which is the pre-arthritic alignment, as shown in Figure 1 [1]. Our surgical strategy adopts the functional alignment (FA) philosophy. Functional alignment is a concept of restoring native knee alignment and functional ligament pattern with adjustments made in component positioning to achieve a better soft tissue balancing in TKA [27]. It stressed the importance of maintaining a balanced gap, joint line obliquity and the final coronal alignment within HKA 174–180°. In one prospective comparisons study, FA group was reported to have better patient outcome and satisfaction with less outliers in implant positioning than MA group [28]. Another study also compares FA and adjusted MA suggesting that patient in the FA group has better KSS with restoration of femoral anatomy and tibia varus. Functional group has slight lateral laxity in knee flexion than extension which resembles the natural ligament pattern [29]. Whereas in study comparing FA to KA, FA group is associated with better flexion gap balancing and less bone resection compared to KA group [30]. In subgroup analysis, Van De Graaf VA et al. had also reported FA has highest proportion of balanced knee which is 96.5% in comparison with 66.4% in KA group and 54.7% in MA group [31].

Our patient has postoperative HKA angle within 6° as planned by FA with robotic assistance. Intraoperative rotation setting was within ER 6° from Posterior Condylar Axis (PCA). Similar joint line obliquity was maintained in comparison to the contralateral limb and knee phenotypes were restored. Medio-lateral flexion and extension gap balance difference was within 1 mm. Self-reported functional outcome was excellent after TKA with large improvement in Knee Society Score (KSS). We advise the preparation of next level of constraint implant or endoprosthesis in anticipating for difficult correction during surgery.

## Limitation

The limitation of our study is small number of patients. However, this is a rare condition due to advances in orthopaedic surgery with less long bone fracture being managed conservatively and hence less malunion cases. Next is on the rotational or axial deformity which is taken into consideration in every case by physical examination of rotational profile and up to 10-degree axial rotation would be accepted for this surgery. However this does not affect the implant positioning internal or external rotation following functional alignment technique. Nonetheless, good result was reported with up to 10° of rotational deformity [16]. We suggest preoperative CT scan from bilateral hip to bilateral ankle using axial cut to accurately determine the rotational abnormality.

## Conclusion

Intraarticular correction by total knee arthroplasty following FA philosophy, with robotic technology assistance, is technically reliable for knee arthritis with extra-articular deformity. It allowed restoration of the lower limb alignment closer to native axis with a balanced knee and produced satisfactory outcomes.

## Data Availability

The data of this study is available from the corresponding author, Wei Cheong Eu, upon reasonable request.
